# Supplementary material for: Impact of AI-Based Post-Processing on Image Quality of Non-Contrast Computed Tomography of the Chest and Abdomen
Source: Diagnostics (Basel). 2024 Mar 13;14(6):612. doi: 10.3390/diagnostics14060612 (PMC10969114; doi:10.3390/diagnostics14060612)
Supplement: Supplementary file 1 [file diagnostics-14-00612-s001.zip › Supplementary data Pixelshine.pdf]

Supplementary data

Supplementary Table S1. Mean relative differences of CT values, noise, SNR and CNR comparing FBP+PS vs. FBP reconstructed thoracal CT. p-values (<0.05) are highlighted.

	Ascending aorta	Pulmonary trunk	Descending Aorta	Lung	Autochthonous muscle	Fat	Air
			**B40 [soft-tissue kernel]**				
**CT FBP vs. FBP+PS**	-0.001+/-0.01 p =0.115	-0.000 +/- 0.01 p=0.684	0.002+/-0.014 p=0.409	-0.001+/-0.00 **p=<0.001**	0.0179+/-0.11 p=0.929	**-0.011 +/- 0.00 p=<0.001**	-0.001+/- 0.00 **p=<0.001**
**Noise FBP vs. FBP+PS**	-0.794+/-0.30 **p=<0.001**	-0.821+/-0.29 **p=<0.001**	-0.765+/-0.27 **p=<0.001**	-0.089+/-0.10 **p=<0.001**	-0.326+/-0.13 **p=<0.001**	-0.347+/- 0.20 **p=<0.001**	-1.400+/- 0.74 **p=<0.001**
**SNR FBP vs. FBP+PS**	0.425+/-0.10 **p=<0.001**	0.436+/-0.10 **p=<0.001**	0.420+/-0.10 **p=<0.001**	0.075+/-0.08 **p=<0.001**	0.256+/-0.11 **p=<0.001**	0.234+/- 0.11 **p=<0.001**	0.53 +/-0.18 **p=<0.001**
**CNR FBP vs. FBP+PS**	0.236+/-0.11 **p=<0.001**	0.236+/-0.11 **p=<0.001**	0.236+/-0.11 **p=<0.001**	0.242+/-0.11 **p=<0.001**	0.235+/-0.11 **p=<0.001**	-	0.242+/- 0.11 **p=<0.001**
			**B70 [lung kernel]**				
**CT FBP vs. FBP+PS**	0.005+/-0.02 p=0.316	-0.038+/-0.21 p=0.054	-0.002+/-0.02 p=0.849	-0.002+/-0.00 **p=<0.001**	0.188+/- 0.85p=0.003	-0.019+/- 0.00 **p=<0.001**	-0.002+0.00 **p=<0.001**
**Noise FBP vs. FBP+PS**	-0.748+/-0.36 **p=<0.001**	-0.759+/-0.35 **p=<0.001**	-0.698+/- 0.259 **p=<0.001**	-0.119+/-0.18 **p=0.002**	--0.370+/-0.13 **p=<0.001**	-0.386+/- 0.27 **p=<0.001**	-0.887+/- 0.60 **p=<0.001**
**SNR FBP vs. FBP+PS**	0.407+/-0.12 **p=<0.001**	0.386+/-0.21 **p=<0.001**	0.396+/-0.11 **p=<0.001**	0.085+/-0.125 **p=0.002**	0.425+/-0.73 **p=<0.001**	0.241+/- 0.13 **p=<0.001**	0.418+/- 0.17 **p=<0.001**
**CNR FBP vs. FBP+PS**	0.245+/-0.13 **p=<0.001**	0.245+/-0.13 **p=<0.001**	0.244+/-0.136 **p=<0.001**	0.255+/-0.13 **p=<0.001**	0.246+/-0.14 **p=<0.001**	-	0.255+/- 0.13 **p=<0.001**

Supplementary Table S2. Mean relative differences of CT values, noise, SNR and CNR comparing FBP+PS vs. FBP, IR+PS vs. IR and FBP+PS vs. IR reconstructed abdominal LD-CT in Site I and Site II. Significant p-values (<0.05) are highlighted.

		Left Liver lobule	Spleen	Descending Aorta	Autochthonous muscle	Fat	Air
				**Site I**			
**FBP vs. FBP+PS**	CT	0.003 +/-0.02 p=0.532	-0.004 +/- 0.02 p=0.191	-0.001+/-0.01 p=0.569	-0.003+/-0.01 p=0.351	-0.01+/- 0.01 **p=<0.001**	0.001+/- 0.00 **p=<0.001**
	Noise	-0.735+/-0.29 **p=<0.001**	-0.692+/-0.35 **p=<0.001**	-0.707+/-0.27 **p=<0.001**	-0.524+/-0.30 **p=<0.001**	-0.535+/- 0.43 **p=<0.001**	-1.723+/- 1.05 **p=<0.001**
	SNR	-0.409+/-0.10 **p=<0.001**	-0.383+/-0.12 **p=<0.001**	-0.400+/-0.10 **p=<0.001**	-0.317+/-0.13 **p=<0.001**	-0.297+/- 0.17 **p=<0.001**	-0.564+/- 0.20 **p=<0.001**

	CNR	-0.300+/-0.17<br>**p=<0.001**	-0.299+/-0.17<br>**p=<0.001**	-0.300+/- 0.17<br>**p=<0.001**	-0.299+/- 0.17<br>**p=<0.001**	-	-0.305+/-<br>0.17<br>**p=<0.001**
**Site II**							
**FBP vs.<br>FBP+PS**	CT	0.002+/-0.12<br>p=0.766	-0.002+/-0.01<br>p=0.293	0.006+/-0.02<br>**p=0.008**	-0.002+/-0.01<br>p=0.152	-0.12+/-<br>0.01<br>**p=<0.001**	-0.001+/-<br>0.00<br>**p=<0.001**
	Noise	-0.860+/-0.28<br>**p=<0.001**	-0.766+/-0.27<br>**p=<0.001**	-0.785+/-0.27<br>**p=<0.001**	-0.611+/-0.24<br>**p=<0.001**	-0.471+/-<br>0.21<br>**p=<0.001**	-1.62+/-<br>0.60<br>**p=<0.001**
	SNR	-0.453+/-0.07<br>**p=<0.001**	-0.421+/-0.08<br>**p=<0.001**	-0.432+/-0.08<br>**p=<0.001**	-0.365+/-0.09<br>**p=<0.001**	-0.299+/-<br>0.09<br>**p=<0.001**	-0.597+/-<br>0.10<br>**p=<0.001**
	CNR	-0.303+/-0.09<br>**p=<0.001**	-0.302+/-0.09<br>**p=<0.001**	-0.303+/-0.09<br>**p=<0.001**	-0.301+/-0.09<br>**p=<0.001**	-	-0.308+/-<br>0.09<br>**p=<0.001**
**IR vs.<br>IR+PS**	CT	0.002+/-0.01<br>p=0.061	0.000+/- 0.01<br>p=0.485	0.008+/- 0.01<br>**p=<0.001**	0.002+/- 0.00<br>**p=0.001**	-0.010+/-<br>0.00<br>**p=<0.001**	-0.001+/-<br>0.00<br>**p=<0.001**
	Noise	-0.309+/-0.15<br>**p=<0.001**	-0.214+/-0.12<br>**p=<0.001**	-0.267+/-0.13<br>**p=<0.001**	-0.129+/-0.10<br>**p=<0.001**	-0.082+/-<br>0.08<br>**p=<0.001**	-0.349+/-<br>0.28<br>**p=<0.001**
	SNR	-0.230+/-0.07<br>**p=<0.001**	-0.170+/-0.07<br>**p=<0.001**	-0.209+/-0.08<br>**p=<0.001**	-0.109+/-0.08<br>**p=<0.001**	-0.061 +/-<br>0.06<br>**p=<0.001**	-0.231+/-<br>0.14<br>**p=<0.001**
	CNR	-0.066+/-0.06<br>**p=<0.001**	-0.065+/-0.06<br>**p=<0.001**	-0.067+/-0.06<br>**p=<0.001**	-0.066+/-0.06<br>**p=<0.001**	-	-0.071+/-<br>0.06<br>**p=<0.001**
**IR vs.<br>FBP+PS**	CT	0.003+/-0.02<br>p=0.626	0.001+/-0.01<br>p=0.719	-0.003+/-0.02<br>p=0.361	-0.002+/-0.01<br>p=0.343	-0.013+/-<br>0.01<br>**p=<0.001**	-0.002+/-<br>0.00<br>**p=<0.001**
	Noise	-0.240+/-0.17<br>**p=<0.001**	-0.163+/-0.17<br>**p=<0.001**	-0.186+/-0.17<br>**p=<0.001**	-0.078+/-0.14<br>**p=0.004**	-0.005+/-<br>0.13<br>p=0.364	-0.888+/-<br>0.43<br>**p=<0.001**
	SNR	0.320+/-0.11<br>**p=<0.001**	0.295+/-0.04<br>**p=<0.001**	0.389+/-0.10<br>**p=<0.001**	0.285+/-0.07<br>**p=0.001**	-0.008+/-<br>0.00<br>p=0.221	-0.170+/-<br>0.03<br>**p=<0.001**
	CNR	0.105+/- 0.02<br>p=0.369	0.107+/-0.02<br>p=0.322	0.116+/-0.02<br>p=0.280	0.107+/-0.02<br>p=0.353	-	-0.017+/-<br>0.00<br>p=0.552
